# Genome-Wide Analysis of Codon Usage Patterns of SARS-CoV-2 Virus Reveals Global Heterogeneity of COVID-19

**DOI:** 10.3390/biom11060912

**Published:** 2021-06-18

**Authors:** Saadullah Khattak, Mohd Ahmar Rauf, Qamar Zaman, Yasir Ali, Shabeen Fatima, Pir Muhammad, Tao Li, Hamza Ali Khan, Azhar Abbas Khan, Ebenezeri Erasto Ngowi, Dong-Dong Wu, Xin-Ying Ji

**Affiliations:** 1 Henan International Joint Laboratory for Nuclear Protein Regulation, School of Basic Medical Sciences, Henan University, Kaifeng 475004, Henan, China; saadullah@henu.edu.cn (S.K.); gataoli@yeah.com (T.L.); azharabbaskhan84@gmail.com (A.A.K.); ebenezerngowi92@gmail.com (E.E.N.); 2 School of Life Sciences, Henan University, Kaifeng 475004, Henan, China; hb7059@wayne.edu or; 3 School of Pharmaceutical Sciences, Wayne State University, Detroit, MI 48202, USA; 4 Department of Bioinformatics, Hazara University, Mansehra 21120, Pakistan; qamarktk348@gmail.com (Q.Z.); hamzaalikhan2211@gmail.com (H.A.K.); 5 National Center for Bioinformatics, Quaid-i-Azam University, Islamabad 45320, Pakistan; yasirkhanqu@gmail.com; 6 Department of Biosciences and Bioinformatics, Capital University of Science and Technology, Islamabad 44000, Pakistan; shabeenfatima53@gmail.com; 7 Henan-Macquarie University Joint Centre for Biomedical Innovation, School of Life Sciences, Henan University, Kaifeng 475004, Henan, China; pir.muhammad786@gmail.com; 8 Department of Biochemistry, Hazara University, Mansehra 21110, Pakistan; 9 School of Stomatology, Henan University, Kaifeng 475004, Henan, China; 10 Kaifeng Key Laboratory of Infection and Biological Safety, School of Basic Medical Sciences, Henan University, Kaifeng 475004, Henan, China

**Keywords:** Coronavirus, SARS-CoV-2, Codon usage bias, COVID-19, heterogeneity of COVID-19, mutational bias, natural selection

## Abstract

The ongoing outbreak of coronavirus disease COVID-19 is significantly implicated by global heterogeneity in the genome organization of severe acute respiratory syndrome coronavirus 2 (SARS-CoV-2). The causative agents of global heterogeneity in the whole genome of SARS-CoV-2 are not well characterized due to the lack of comparative study of a large enough sample size from around the globe to reduce the standard deviation to the acceptable margin of error. To better understand the SARS-CoV-2 genome architecture, we have performed a comprehensive analysis of codon usage bias of sixty (60) strains to get a snapshot of its global heterogeneity. Our study shows a relatively low codon usage bias in the SARS-CoV-2 viral genome globally, with nearly all the over-preferred codons’ A.U. ended. We concluded that the SARS-CoV-2 genome is primarily shaped by mutation pressure; however, marginal selection pressure cannot be overlooked. Within the A/U rich virus genomes of SARS-CoV-2, the standard deviation in G.C. (42.91% ± 5.84%) and the GC3 value (30.14% ± 6.93%) points towards global heterogeneity of the virus. Several SARS-CoV-2 viral strains were originated from different viral lineages at the exact geographic location also supports this fact. Taking all together, these findings suggest that the general root ancestry of the global genomes are different with different genome’s level adaptation to host. This research may provide new insights into the codon patterns, host adaptation, and global heterogeneity of SARS-CoV-2.

## 1. Introduction

Severe acute respiratory syndrome coronavirus 2 (SARS-CoV-2) is a non-segmented positive sense, enveloped, single-stranded RNA virus that belongs to genus Coronavirus, order Nidovirales, and family Coronaviridae [1]. It is the seventh member of the Coronaviridae family that infects humans. Among the prior six, NL63, HKU1, 229E and OC43 are known to show mild symptoms, while MERS-CoV, SARS-CoV, and recently emerged SARS-CoV-2 are associated with severe diseases [2]. SARS-CoV-2 propagates a respiratory and gastrointestinal infection referred to as coronavirus disease 2019 (CoVID-19), an ongoing pandemic [3]. The symptoms include dry cough, fever, fatigue, dyspnea, and lymphopenia with occasional human complications like severe acute respiratory syndrome (SARS), pneumonia, and even death [4]. The SARS-CoV-2 genome ranges from 27–32 kb and carries a unique replication strategy [5]. The genome comprises of several open reading frames like ORF1ab, ORF3a, ORF6, ORF7a, ORF7b ORF8, and ORF10 that codes for both structural proteins like spike protein, replicase polyprotein, envelope protein, accessory proteins, nucleocapsid proteins, and other Non-structural proteins (NSP) [6,7,8].

However, the recent global spread of SARS-COV-2, with surging transmissions in many parts of the world and steep declines in others, has raised fundamental questions about the viral genome’s evolution and adaptation with the host genome [9]. The counter causes of global heterogeneity are variation in host immune systems, mutations, deletions, recombination, genetic drift, and the founder’s impact [10]. According to some studies, adaptations at nucleotide [1] and amino acid [4] regions, as well as heterogeneity found in structural proteins, significantly spike proteins, are possible causes of global variable transmission patterns [11,12,13]. The evolution of the viral genome, transmissibility, and virulence can be linked to intra-host viral evolution after infection [13,14]. Virus infectivity is directly affected by deleted variants of accessory proteins and non-structural proteins [15,16,17]. However, both reports are limited to comparing only a few genomes from a few countries and concentrating primarily on structural proteins. Codon usage research will allow us to understand the global heterogeneity of SARS-CoV-2, the evolution of its genome, and its adaptation to the host genome. In the translation process, different strains use different codons [13,18,19,20]. The translation process is primarily driven by synonymous codons [21], which code for the same amino acids but at different frequencies [22,23]. Codon usage bias (CUB) is common in eukaryotes, prokaryotes, viruses, and other genes in the same organism [24]. Codon usage patterns are more pronounced in highly expressed genes than in lower expressed genes [25]. The codon usage pattern is influenced by transcriptional factors, translation, G.C. content, secondary motifs, and gene expression level [23]. Natural selection and mutational pressure, on the other hand, are the primary driving forces [26,27]. Several studies have concluded that mutational pressure is the primary driver of codon usage patterns in SARS-CoV-2 [23,28,29,30,31].

In contrast to eukaryotes and prokaryotes, viral genomes have unique characteristics such as host dependence for replication, synthesis, and protein transmission. These essential characteristics affect virus development, adaptation, survival, and host immune system disregard [32,33]. Understanding the codon usage pattern can thus provide valuable insights into the evolutionary process, host adaptation, disregard for the host immune system, viral pathogenesis, and global heterogeneity of the SARS-CoV-2 virus [12,14,20,29]. For this purpose, we have characterized the codon usage patterns among sixty SARS-CoV-2 viral strains to unravel the role of codon architecture in viral pathogenesis. The possible causes of this newly emerging coronavirus’s global heterogeneity and its adaptation to host and insights into the viral lineages were gained using bioinformatics approaches.

## 2. Material and Methods:

### 2.1. Acquisition of Data

The whole-genome sequences of 60 SARS-CoV-19 isolates reported worldwide were retrieved from the National Center for Biotechnology Information (NCBI) (https://www.ncbi.nlm.nih.gov (accessed on 16 February 2021)). The sequences were selected based on geographical distribution across five continents, transmission rate, and isolation methods. The CDS sequences were concatenated using the Artemis genome browser [34] and aligned through ClustalW [35]. The codon usage data of the SARS-CoV-2 host, Homo Sapiens, was retrieved from the codon usage database (https://www.kazusa.or.jp/codon/ (accessed on 23 February 2021)).

### 2.2. Nucleotide Composition of SARS-CoV-2

Nucleotide composition analysis of CDS sequences of 60 SARS-CoV-2 confines was helped out through the Artemis genome program [34], codonW (http://codonw.sourceforge.net//culong.html (accessed on 5 January 2021)) and Emboss pilgrim [36]. The genome-level individual frequencies of the nucleotide (A, T, G and C,) were checked during composition analysis. This was followed by finding the collective frequencies of A.U. and G.C. throughout the genomes and the occurrence of A, U, G and C at 1st, 2nd, and third places of codons. The mean value of AU3, GC12 and GC3 was recorded for all the strains. Herein, AUG and UGG bearing no synonymous codons, while UGA, UAG, and UAA stop codons were neglected in the protocol.

### 2.3. Codon Preference Characteristics

The overall relative synonymous codon usage (RSCU) is the proportion of codons’ observed recurrence compared to the regular recurrence of codons under uniform synonymous codon utilization. An RSCU value equivalent to 1 reflects that codon usage is not biased. RSCU values under 1.0 happen when the observed recurrence is not precisely the regular recurrence [37]. The codon preferences of SARS-CoV-2 were calculated and compared with other hosts like humans, dogs, cats and cattle to show the codon preferences of natural and other hosts.

### 2.4. Analysis of Codon Usage in SARS-CoV-2

CodonW (available at http://sourceforge.net/projects/codonw (accessed on 5 January 2021)) was employed to perform codon usage bias analysis by calculating relative synonymous codon usage RSCU values [38]. An adequate number of codons ENC plot analysis was also performed to reveal the usage bias pattern in the CDS regions of SARS-CoV-2 [39]. To carry out the correlation studies and demonstrate the adaptation of SARS-CoV-2 to its host, the Codon adaptation index of all the strains was measured against the reference human genome’s codons usage pattern [40].

### 2.5. ENC-Plot Analysis

An ENC plot will shed light on the ENC relationship and the G.C. content at the third codon location (GC3). This method demonstrates gene codon usage bias. It is commonly used to assess the extent of a gene’s codon usage bias. To determine the correlation, the predicted ENC values for the corresponding GC3 were calculated using Singh et al. process [41]. There is a solid line that represents the expected curve where if the strains lie close or on the line, represents mutational pressure being the driving force or if lower, shows selection pressure as well in addition to mutational pressure.

### 2.6. Neutral Evolution Analysis

Neutral evolution analysis or neutrality plot analysis is used to determine the factors that affect codon usage preference [42]. It was used to evaluate the mutation-selection equilibrium in shaping the codon usage bias. Using GC3 as a horizontal coordinate and GC12 as a vertical coordinate, the GC3 and GC12 contents were plotted with a regression line to determine how mutational pressures played a role in forming codon usage bias instead of natural selection [42].

### 2.7. Codon Adaptation Analysis

Codon usage similarities of host *Homo Sapiens* and SARS-CoV-2 genomes were quantified using codon adaptation index (CAI) [40]. CAI analysis can reveal respective codons for those amino acids that are more efficient for translation and are highly expressed genes. CAI values ranges between 0.0 and 1.0, where higher CAI values depict higher gene expression potential and vice versa. Further, values that are close to one indicate that codons with higher RSCU values are used in the CDS sequences. The host synonymous codon usage bias data was extracted from the codon usage bias database (http://www.kazusa.or.jp/codon/ (accessed on 23 February 2021)), compiled from 93487 CDS sequences of Homo Sapiens. Wilcoxon & Mann Whitney test was utilized to identify statistically significant CAI values [43]. To show that the CAI value’s significance is solely due to codon usage pattern preferences, the expected CAI (eCAI) was calculated at a confidence interval of 95% [44].

### 2.8. Correspondence Analysis (COA)

Correspondence analysis is a multivariate statistical analysis that is used to detect variable and sample relationships. Correspondence analysis (COA) is a broadly used statistical method to analyze multiple factors and their influences on a specific component. COA displays sets of rows and columns in a particular data set [45]. This approach helps to reflect the trend of strain change directly. The codonW program was used in this study to perform COA based on RSCU values.

### 2.9. Phylogenetic Analysis

Phylogenetic analysis was performed to depict the genetic diversity and evolutionary relationships among SARS-CoV-2 strains retrieved from NCBI. The sequences were aligned using the ClustalW program [46]. The phylogenetic tree was constructed using Mega 7, utilizing the maximum likelihood method [47]. In the present study, we performed comprehensive analyses of codon usage and composition of SARS-CoV-2 strains and checked the possible leading evolutionary element of the biases found.

## 3. Results and Discussion

### 3.1. Nucleotide Composition Analysis of SARS-CoV-2

**Figure 1:**
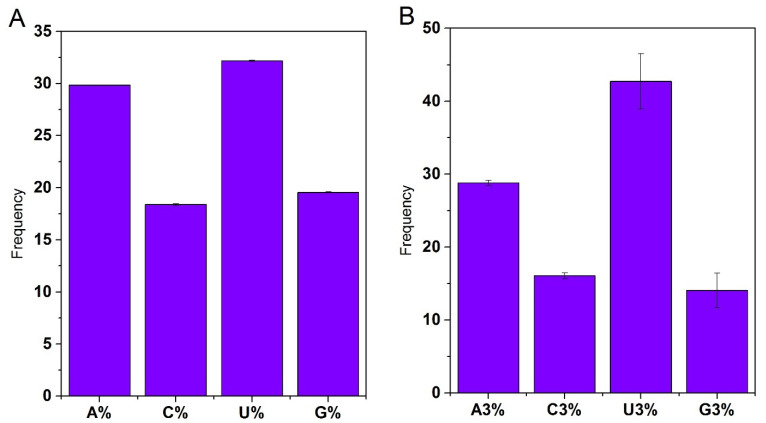
Nucleotide content distribution of CDS sequences of SARS-CoV-2 in percentage. (**A**) The average composition of individual bases A, C, U, G. among 60 strains. (**B**) A, C, U, and G frequency at the third position of the codons.

Nucleotide composition being the major force in affecting codon usage pattern was measured to evaluate its impact on the codon usage pattern of CDS sequences of SARS-CoV-2 [48]. The composition frequency and trend of each nucleotide were U (32.19% ± 0.05) > A (29.85% ± 0.02) > G (19.56% ± 0.05) > C (18.39% ± 0.07), which is consistent with the trend in other Coronaviruses like SARS and MERS [29] which manifestly indicate U codon being the more frequent one throughout SARS-CoV-2 genome (Supplementary Table S1, Figure 1A) However, these trends differ from other viruses like RSV which follows A > U > G > C and H1N1 and H3N2 order A > G > U > C [29]. AU’s mean values (62.04% ± 0.04) and GC (37.96% ± 0.04) emphasize the CDS genome as AU-rich genome (Supplementary Table S1). The nucleotide composition of codons at third position U3 (42.73% ± 3.93) > A3 (28.80% ± 4.03) > C3 (16.08% ± 2.21) > G3 (14.04% ± 4.90 showed inconsistent trend against SARS-CoV and MERS which follows U3 > A3 > G3 > C3 [29]. Interestingly, this trend is also inconsistent with the overall trend of bases in the genome (Supplementary Table S1, Figure 1B). However, the higher AU content results align with other RNA viruses like SARS, showing high A.U. content and preferred A/U ending codons [49]. The GC12 value of 42.91% ± 5.84 and GC3 value of 30.14% with a standard deviation SD of 6.93 indicates the biasness in the codon usage pattern manifesting global heterogeneity (Supplementary Table S1).

### 3.2. Relative Synonymous Codon Usage (RSCU) Analysis

**Figure 2:**
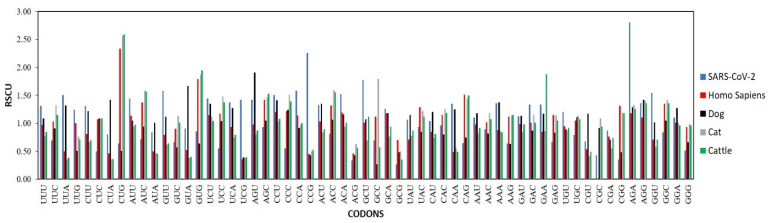
Comparisons of RSCU values of SARS-CoV-2 virus with its host humans, dog, cat, and cattle. The dog, cat, and cattle RSCU is shown as calculated by Dutta et al. [53].

The RSCU values for 60 strains were calculated and compared to the host’s human, dog, cat, and cattle genomes to get insights into the codon usage bias pattern of the SARS-CoV-2 viral genome. The results implicitly demonstrated that all 18 frequent codons were A/U ended, which shows SARS-CoV-2 genomes, higher bias towards A/U than G/C. These results are consistent with other viral genomes like avian rotaviruses, equine influenza viruses, and Crimean-Congo hemorrhagic fever virus [49]. In contrast, all the ten under preferred codons were G/C ended, which manifests that the SARS-CoV-2 viral genome is mostly under mutational pressure, which helps them avoid the host’s innate immunity [50] (Table 1, Figure 2). 14 out of 59 codons were similar in both the host and viral genome, which improves the translation efficiency of the SARS-CoV-2 within humans like BTV viruses which have 9/59 similar codons with its host Bos Taurus [51,52] (Table 1, Figure 2). Analysis of RSCU values of SARS-CoV-2 and its different hosts uncovered the codon preferences of SARS-CoV-2, human, dog, cat, pig, horse, and cattle (Table 1). The average RSCU of SARS-CoV-2 was compared to that of its regular (human) and accidental (dog) hosts along with other animal species, which revealed that the codon preference of SARS-CoV-2 and its hosts (natural, accidental, and other) are not similar (Figure 2) [53].

### 3.3. Codon Usage Bias Analysis of SARS-CoV-2 Genomes

**Figure 3:**
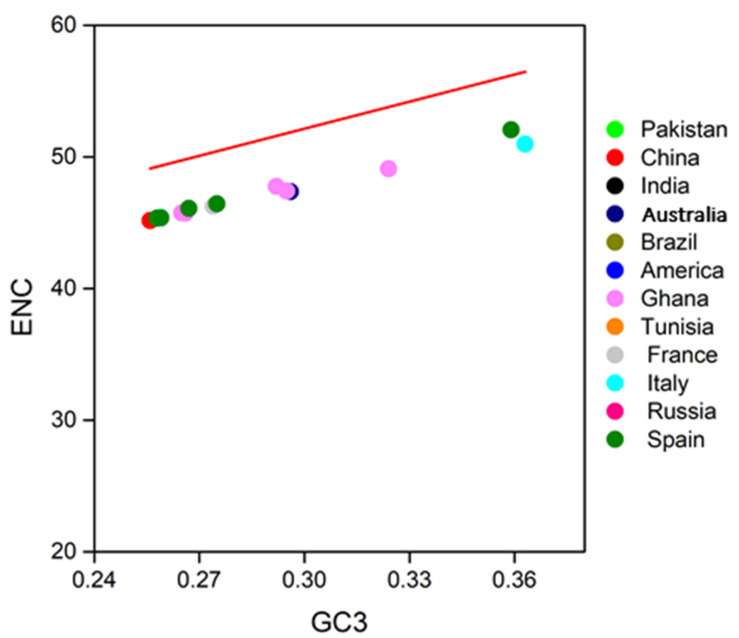
ENC versus GC3 plots of 60 SARS-CoV-2 isolates are represented in different color schemes.

To identify the magnitude of codon usage pattern among CDS sequences of SARS-CoV-2 virus, we have calculated and analyzed all the 60 strains’ ENC values. The ENC values ranged from 45.17 of a Chinese strain MT135043.1 to 52.06 of a Spanish strain MT233522.1 with a mean value of 45.80 and a standard deviation of 1.27 (Supplementary Table S2). This value is significantly lower than the mean ENC value of other coronaviruses including BCoV (52.10 ± 2.36), BuCoV HKU11 (51.41 ± 1.85), ECoV (49.31 ± 4.02), FIPV (51.56 ± 1.99) and HCoV-229E (50.29 ± 3.62), among others, which indicates that SARS-CoV-2 uses a relatively lower set of synonymous codons [54,55]. ENC versus GC3 plot was constructed to show that the codon usage pattern of SARS-CoV-2 is mainly under mutational bias (Supplementary Table S3, Figure 3). All the values lie significantly lower to the solid line demonstrate that mutational pressure is not the single factor shaping the codon usage bias. However, other factors such as natural selection are likely to determine the selective constraints on the codon usage bias in 60 strains of SARS-CoV-2 Figure 3. These results are consistent with recently reported SARS-CoV-2 codon usage pattern characterization [20]. However, to show up to which extent both the major driving forces influence the codon usage pattern, the GC12 and GC3 neutrality plot was constructed.

### 3.4. Neutrality Plot

**Figure 4:**
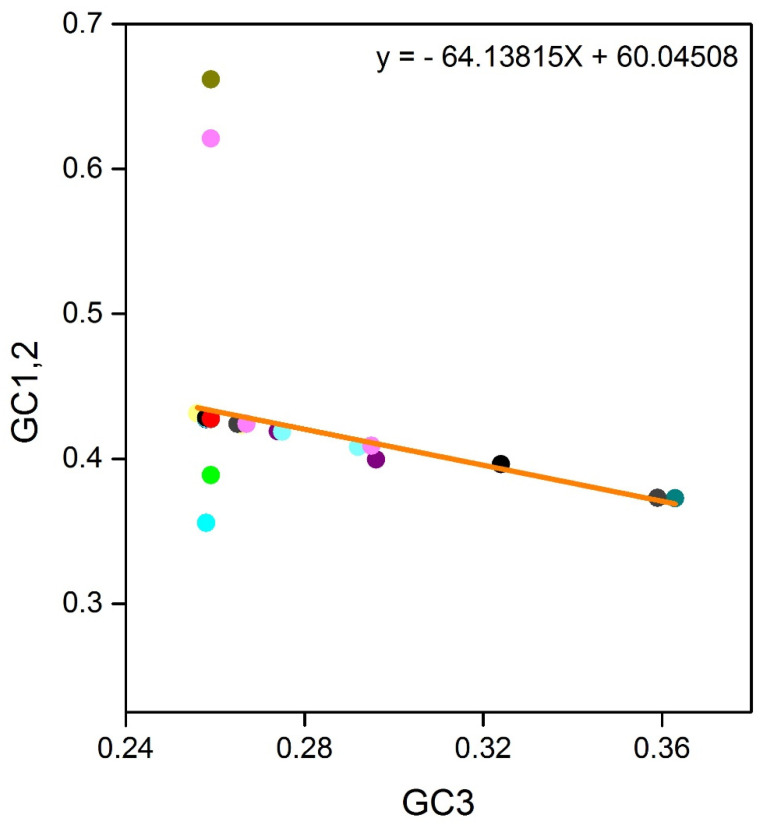
Neutrality plot analysis (GC12 vs. GC3) plots of 60 SARS-CoV-2 isolates are represented in different color schemes. GC12 stands for the average value of G.C. contents at the first and second positions of the codons (GC1 and GC2), while GC3 refers to the G.C. contents at the third codon position.

The degree of Mutation bias and selection pressure was measured through the Neutrality plot between GC12 and GC3 using the Pearson correlation method. The neutrality plot r = −0.31 and *p* > 0.01 indicates that both mutation pressure and natural selection drive the codon usage patterns of SARS-CoV-2. Our analysis also confirmed that most SARS-CoV-2 genomes were present along the unity slope, indicating that the SARS-CoV-2 genome is essentially under mutational pressure. Some of the points were scattered away from the line marking the translational force also marginally but significantly drove the codon usage biases in SARS-CoV-2 (Supplementary Table S4, Figure 4). Our results are consistent with the previously reported results for SARS-CoV-2 [20,29] but inconsistent with some reports [53]. This reconfirms our statement that analysis on a small sample size may result in false positives, so we analyzed a more diverse dataset of 60 genomes.

### 3.5. Codon Adaptation Analysis

The codon adaptation index analysis was performed to demonstrate the adaptation of the SARS-CoV-2 strains to their host. CAI values are used to determine the level of expression of pathogen proteins in the host and the adaptation of a virus to a host. Sequences with higher CAI values are considered more adapted to a particular host than those with low values. The CAI value of SARS-CoV-2 concerning humans (0.70 ± 0.01) is higher than 0.62 ± 0.01, 0.59 ± 0.00, and 0.61 ± 0.01 concerning the dog, cattle, and cat. These values show the higher adaptation of SARS-CoV-2 to the human environment compared to other hosts. (Supplementary Table S5, Figure S1). The higher tendency of the human CAI value shows that selection pressure from humans can affect the codon patterns of SARS-CoV-2 which have allowed it to use the translation source of humans more efficiently as in line with Marburg virus adaptation to the human host [18] (Supplementary Figure S1). The higher average CAI values of humans compared to dogs, cats, and cattle observed in the present study indicated that dogs and other hosts are less susceptible to COVID 19 than humans. However, the cross-transmission of SARS-CoV-2 between humans and dogs, cats, and cattle, has not been well-understood [53]. Furthermore, to validate the statistical significance, the expected CAI (e-CAI) values were computed for SARS-CoV-2, humans, dogs, cattle, and cat codon usage sets by generating 500 uneven sequences with similar nucleotide contents and amino acid composition as the sequences of interest. The e-CAI values of 0.75, 0.66, 0.61, and 0.65 of humans, dogs, cattle, and cats, respectively, revealed that the generated sequences had a normal distribution.

### 3.6. COA Analysis

**Figure 5:**
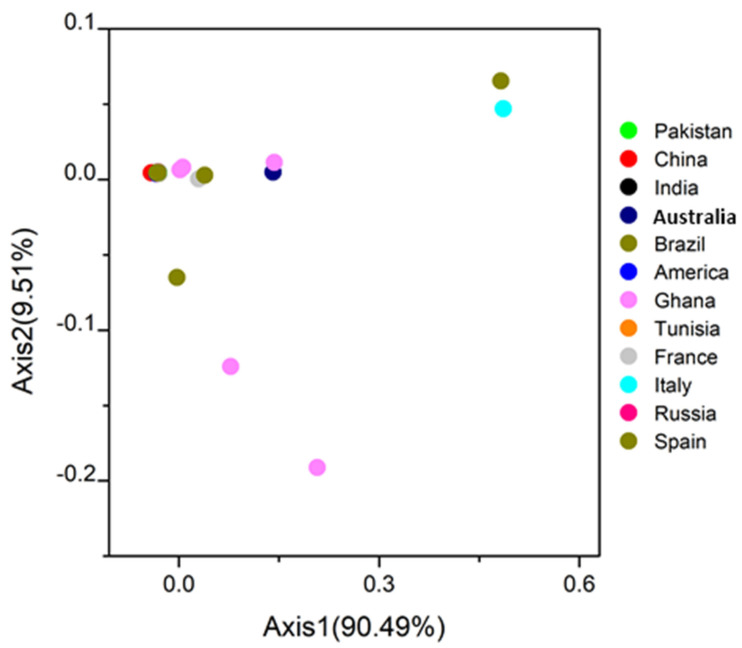
Variation analysis in SARS-CoV-2 genomes: based on the RSCU values, all the strains are plotted in the variance plane. The first and second principal axes represent different geographical origins. Each point represents a strain and shows in different colors.

The sixty strains of SARS-CoV-2 were plotted into clusters based on their RSCU values. All the SARS-CoV-2 strains were clustered into two major groups and two minor clusters, while some were found scattered. The first significant cluster has SARS-CoV-2 strains from Australia, Brazil, China, France, Ghana, India, Italy, Pakistan, Spain, and Tunisia. The second considerable cluster has strains from America, Australia, India, Italy, Pakistan, Russia, and Tunisia. The first small cluster has strains from Brazil, India, Italy, and Russia, while the second has strains from France, Spain, and Russia. There were two scattered strains from Ghana, two from Australia, and one from Italy Figure 5. These results suggested that geographical locations play an essential role in the SARS-CoV-2 evolutionary process and a synonymous codon usage pattern. Besides, it is also highlighted that each infected country has emerged from more than one viral genetic lineage, which depicts those geographic locations have a crucial role in shaping codons [56]. Some countries have distinct genomes, probably due to viral transmission through international trade, human traveling, or bird migration, as in Crimean-Congo [56].

### 3.7. Phylogenetic Analysis

**Figure 6:**
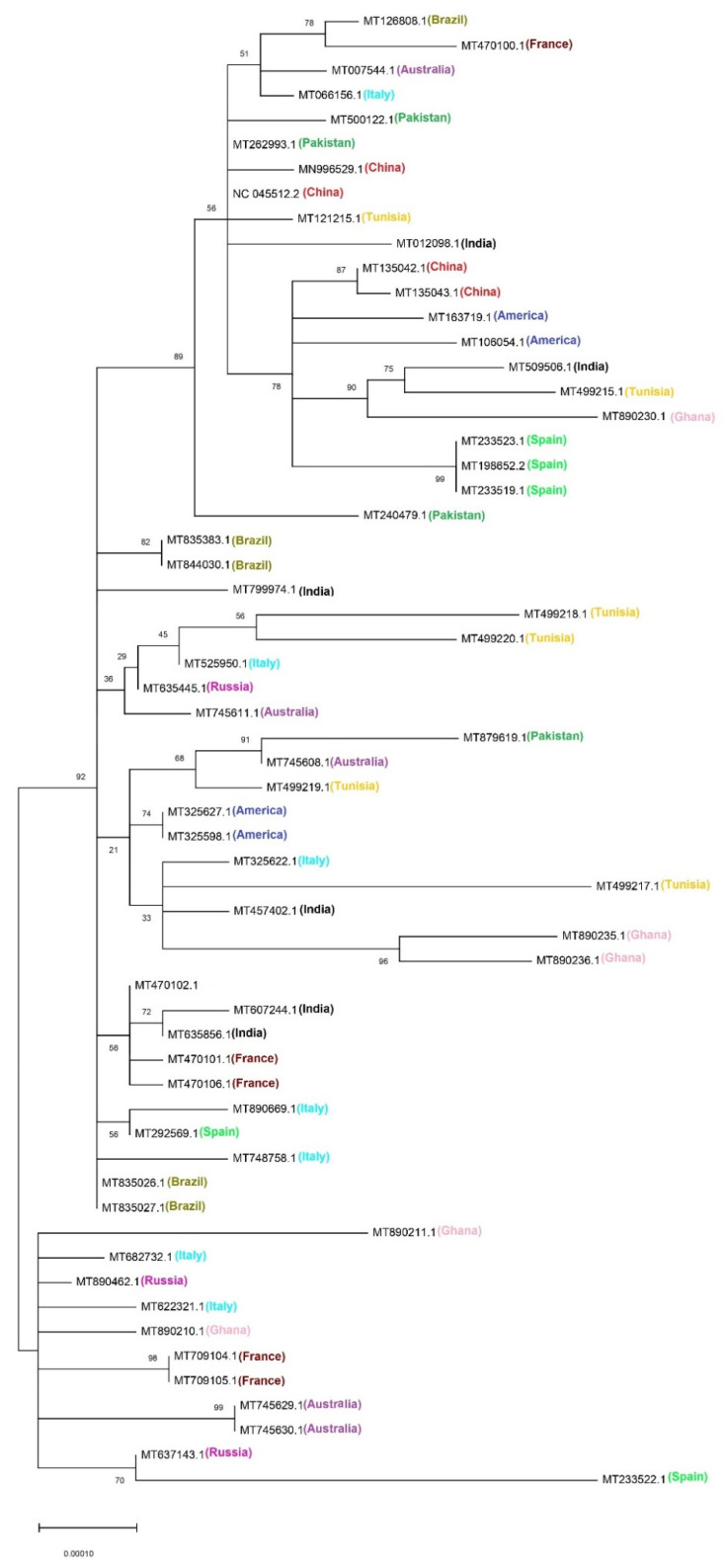
Phylogenetic tree based on the whole genome sequences of 60 SARS-Cov-2 strains. The tree was generated by the maximum likelihood (ML) method using the MUSCLE online tool.

Phylogenetic relationships of sixty SARS-CoV-2 CDS genomes were depicted from a Phylogenetic tree. Very high diversity was found in some strains like MT745629.1 and MT745630.1 of Australia, MT89210.1 and MT890211.1 of Ghana, and MT682732.1, MT622321.1 of Italy. Some strains from France (MT709104.1, MT709105.1), Russia (MT890462.1, MT637143.1), and Spain (MT233522.1) though distinct but were found in the same clade. Apart from these, the remaining strains were divided into two major clades. A few strains of the same country were present in different clades, which depict that SARS-CoV-2 can arise from other viral lineages (Figure 6). Like the diversity found in Ghana’s strains, the various evolution in different countries points towards the founder’s effect in the SARS-CoV-2 genome. Moreover, it is also noticed that geographical locations may play a role in viral evolution.

## 4. Conclusions

Based on our findings, we conclude that the SARS-CoV-2 genome is shaped by a relatively low codon usage bias that is primarily motivated by mutational pressure but is also influenced by translation selection, which cannot be overlooked. Some countries’ SARS-CoV-2 strains vary slightly due to different viral lineages. The better codon adaptation with humans can help explain the extensive spreadable nature of the virus. This research makes an essential contribution to the understanding of coronaviruses. Moreover, it is vital to conduct a large-scale comparative analysis of the codon patterns to reduce false positives.

## Figures and Tables

**Table 1:** The Relative synonymous codon usage (RSCU) value of 59 codons encoding 18 amino acids of SARS-CoV-2 virus with their hosts Homo sapiens, dog, cat, and cattle’s RSCU values [53].

AA	Codon	SARS-CoV-2	Human	Dog	Cat	Cattle	AA	Codon	SARS-CoV-2	Human	Dog	Cat	Cattle
Phe	UUU	**1.31**	0.97	**1.09**	0.77	0.85	Ala	GCU	**1.78**	**1.01**	**1.07**	0.69	**1.12**
	UUC	0.69	**1.03**	0.91	**1.33**	**1.15**		GCC	0.69	**1.12**	**0.27**	**1.79**	**0.57**
Leu	UUA	**1.51**	**0.50**	**1.32**	**0.35**	**0.38**		GCA	**1.26**	**1.18**	**1.18**	0.76	0.94
	UUG	**1.25**	**1.00**	**0.51**	0.76	0.71		GCG	**0.27**	0.7	**0.48**	**0.50**	**0.35**
	CUU	**1.31**	0.81	**1.22**	0.67	0.70	Tyr	UAU	**1.06**	0.71	**1.15**	0.78	0.87
	CUC	**0.50**	**1.07**	**1.09**	**1.09**	**1.09**		UAC	0.93	**1.29**	0.85	**1.22**	**1.12**
	CUA	0.80	**0.46**	**1.42**	**0.36**	**0.36**	His	CAU	**1.04**	0.85	**1.2**	0.74	0.81
	CUG	0.63	**2.33**	**0.51**	**2.57**	**2.59**		CAC	0.96	**1.15**	0.80	**1.26**	**1.18**
Iie	AUU	**1.44**	**1.13**	**1.05**	0.95	0.98	Gln	CAA	**1.35**	**0.49**	**1.25**	**0.56**	**0.49**
	AUC	0.72	**1.37**	0.94	**1.58**	**1.57**		CAG	0.65	**1.51**	0.75	**1.44**	**1.50**
	AUA	0.84	**0.50**	**1.01**	**0.47**	**0.45**	Asn	AAU	**1.10**	0.98	**1.18**	0.82	0.92
Val	GUU	**1.58**	0.79	**1.12**	0.62	0.64		AAC	0.90	**1.02**	0.82	**1.18**	**1.07**
	GUC	0.66	0.90	**0.57**	**1.13**	**1.01**	Lys	AAA	**1.36**	0.88	**1.37**	0.86	0.84
	GUA	0.91	**0.52**	**1.67**	**0.38**	**0.40**		AAG	0.64	**1.12**	0.63	**1.14**	**1.15**
	GUG	0.85	**1.79**	0.64	**1.87**	**1.95**	Asp	GAU	**1.13**	0.99	**1.13**	0.84	0.98
Ser	UCU	**1.44**	**1.15**	**1.35**	**1.12**	**1.04**		GAC	0.87	**1.01**	0.87	**1.16**	**1.01**
	UCC	**0.55**	**1.17**	**1.04**	**1.48**	**1.37**	Glu	GAA	**1.33**	0.85	**1.17**	0.86	**1.88**
	UCA	**1.37**	0.93	**1.27**	0.74	0.79		GAG	0.67	**1.15**	0.83	**1.14**	**1.05**
	UCG	**0.28**	**0.36**	**0.39**	**0.38**	**0.39**	Cys	UGU	**1.21**	0.95	0.89	0.87	0.92
	AGU	**1.42**	0.98	**1.91**	0.8	0.87		UGC	0.79	**1.05**	**1.11**	**1.13**	**1.07**
	AGC	0.93	**1.42**	**1.05**	**1.47**	**1.53**	Arg	CGU	0.68	**0.54**	**1.17**	**0.41**	**0.49**
Pro	CCU	**1.50**	**1.20**	**1.41**	**1.03**	**1.08**		CGC	**0.43**	**1.11**	0.92	**1.09**	0.94
	CCC	**0.56**	**1.22**	**1.24**	**1.51**	**1.39**		CGA	0.87	0.76	0.71	**0.55**	0.74
	CCA	**1.58**	**1.14**	0.92	0.97	**1.00**		CGG	**0.35**	**1.31**	**0.48**	**1.19**	**1.18**
	CCG	**0.14**	**0.45**	**0.43**	**0.50**	**0.53**		AGA	**2.81**	**1.18**	**1.29**	**1.33**	**1.26**
Thr	ACU	**1.32**	**1.03**	**1.35**	0.84	0.89		AGG	**1.36**	**1.1**	**1.42**	**1.41**	**1.36**
	ACC	0.82	**1.32**	**1.06**	**1.59**	**1.55**	Gly	GGU	**1.54**	0.71	**1.02**	**0.58**	0.71
	ACA	**1.52**	**1.19**	**1.16**	0.94	**1.01**		GGC	0.84	**1.35**	**1.05**	**1.42**	**1.36**
	ACG	**0.34**	**0.46**	**0.43**	0.63	**0.56**		GGA	**1.10**	**1.01**	**1.27**	**1.01**	**0.96**
								GGG	**0.52**	0.93	0.66	0.99	0.96

AA, amino acid; SARS-CoV-2, severe acute respiratory syndrome coronavirus 2; RSCU. Relative Synonymous codon usage: Underlined codons represent optimal codons of SARS-CoV-2; Blue color represents most favored codons by both host and SARS-CoV-2 having RSCU value greater than 1, pink and dark green colors represent under preferred (RSCU < 0.6) and over preferred (RSCU > 1.6) codons, respectively.

## Data Availability

All data are available with proper figures, tables, captions, legends in the manuscript, and supplementary files.

## Supplementary Materials

The following are available online at https://www.mdpi.com/article/10.3390/biom11060912/s1. Table S1: ENC Values of sixty SARS-CoV-2 Strains around the globe with mean ENC value, Standard deviation and Maximum and minimum ENC value. Table S2: ENC and GC3 values of all the sixty strains of SARS-CoV-2 viruses from around the globe with their mean and standard deviation. Table S3: Neutrality plot (GC3 and GC12) value comparison of all the sixty strains of SARS-CoV-2. Table S4: CAI values of sixty strains of SARS-CoV-2 with host humans. Figure S1: Mean CAI value and Expected CAI value of SARS-CoV-2 strains against its host Humo sapiens.
