# Supplementary material for: Genome-Wide Analysis of Codon Usage Patterns of SARS-CoV-2 Virus Reveals Global Heterogeneity of COVID-19
Source: Biomolecules. 2021 Jun 18;11(6):912. doi: 10.3390/biom11060912 (PMC8233742; doi:10.3390/biom11060912)
Supplement: Supplementary file 1 [file biomolecules-11-00912-s001.zip › biomolecules-1209988-supplement.pdf]

Supplementary Table S1: *ENC Values of sixty SARS-CoV-2 Strains around the globe with mean ENC value, Standard deviation and Maximum and minimum ENC value.*

**S. No**	**Accession Number**	**Country**	**ENC**	**S. No**	**Accession Number**	**Country**	**ENC**
1	MT500122.1	Pakistan	45.37	31	MT890210.1	Ghana	45.73
2	MT879619.1	Pakistan	45.33	32	MT890211.1	Ghana	47.77
3	MT262993.1	Pakistan	45.38	33	MT890230.1	Ghana	47.41
4	MT240479.1	Pakistan	45.36	34	MT890235.1	Ghana	45.74
5	MT135042.1	China	45.38	35	MT890236.1	Ghana	49.1
6	MT135043.1	China	45.37	36	MT499215.1	Tunisia	45.37
7	MT121215.1	China	45.17	37	MT499217.1	Tunisia	45.35
8	MN996529.1	China	45.36	38	MT499218.1	Tunisia	45.37
9	NC_045512.2	China	45.38	39	MT499219.1	Tunisia	45.36
10	MT799974.1	India	45.37	40	MT499220.1	Tunisia	45.37
11	MT607244.1	India	45.37	41	MT470100.1	France	45.37
12	MT635856.1	India	45.37	42	MT470101.1	France	45.37
13	MT457402.1	India	45.36	43	MT470102.1	France	45.37
14	MT012098.1	India	45.39	44	MT709104.1	France	46.26
15	MT509506.1	India	45.35	45	MT709105.1	France	45.38
16	MT007544.1	Australia	45.41	46	MT470106.1	France	45.36
17	MT745608.1	Australia	45.36	47	MT066156.1	Italy	50.98
18	MT745611.1	Australia	45.38	48	MT525950.1	Italy	45.38
19	MT745629.1	Australia	45.36	49	MT622321.1	Italy	45.37
20	MT745630.1	Australia	47.37	50	MT682732.1	Italy	45.39
21	MT126808.1	Brazil	45.39	51	MT748758.1	Italy	45.39
22	MT835026.1	Brazil	45.38	52	MT890669.1	Italy	45.38
23	MT835027.1	Brazil	45.38	53	MT635445.1	Russia	45.38
24	MT835383.1	Brazil	45.38	54	MT637143.1	Russia	45.37
25	MT844030.1	Brazil	45.38	55	MT890462.1	Russia	45.38
26	MT163719.1	USA	45.37	56	MT198652.2	Spain	46.43
27	MT106054.1	USA	45.38	57	MT233519.1	Spain	46.1
28	MT325622.1	USA	45.36	58	MT233522.1	Spain	52.06
29	MT325627.1	USA	45.37	59	MT233523.1	Spain	45.36
30	MT325598.1	USA	45.37	60	MT292569.1	Spain	45.38
Mean				45.80			
Std				1.27			
Range				Max=52.06		Min=45.1	

Supplementary Table S2: *ENC and GC3 values of all the sixty strains of SARS-CoV-2 viruses from around the globe with their mean and standard deviation.*

S. No	Accession Number	Country	ENC	GC3s	ENC exp
1	MT500122.1	Pakistan	45.37	0.26	49.32
2	MT879619.1	Pakistan	45.33	0.26	49.25
3	MT262993.1	Pakistan	45.38	0.26	49.32
4	MT240479.1	Pakistan	45.36	0.26	49.32
5	MT135042.1	China	45.38	0.26	49.32
6	MT135043.1	China	45.37	0.26	49.32
7	MT121215.1	China	45.17	0.26	49.10
8	MN996529.1	China	45.36	0.26	49.32
9	NC_045512.2	China	45.38	0.26	49.32
10	MT799974.1	India	45.37	0.26	49.32
11	MT607244.1	India	45.37	0.26	49.32
12	MT635856.1	India	45.37	0.26	49.32
13	MT457402.1	India	45.36	0.26	49.32
14	MT012098.1	India	45.39	0.26	49.32
15	MT509506.1	India	45.35	0.26	49.25
16	MT007544.1	Australia	45.41	0.26	49.32
17	MT745608.1	Australia	45.36	0.26	49.25
18	MT745611.1	Australia	45.38	0.26	49.32
19	MT745629.1	Australia	45.36	0.26	49.25
20	MT745630.1	Australia	47.37	0.30	52.02
21	MT126808.1	Brazil	45.39	0.26	49.32
22	MT835026.1	Brazil	45.38	0.26	49.32
23	MT835027.1	Brazil	45.38	0.26	49.32
24	MT835383.1	Brazil	45.38	0.26	49.32
25	MT844030.1	Brazil	45.38	0.26	49.32
26	MT163719.1	USA	45.37	0.26	49.32
27	MT106054.1	USA	45.38	0.26	49.32
28	MT325622.1	USA	45.36	0.26	49.32
29	MT325627.1	USA	45.37	0.26	49.32
30	MT325598.1	USA	45.37	0.26	49.32
31	MT890210.1	Ghana	45.73	0.27	49.85
32	MT890211.1	Ghana	47.77	0.29	51.74
33	MT890230.1	Ghana	47.41	0.30	51.95
34	MT890235.1	Ghana	45.74	0.27	49.77
35	MT890236.1	Ghana	49.1	0.32	53.93
36	MT499215.1	Tunisia	45.37	0.26	49.32
37	MT499217.1	Tunisia	45.35	0.26	49.25
38	MT499218.1	Tunisia	45.37	0.26	49.32
39	MT499219.1	Tunisia	45.36	0.26	49.25
40	MT499220.1	Tunisia	45.37	0.26	49.32
41	MT470100.1	France	45.37	0.26	49.32

42	MT470101.1	France	45.37	0.26	49.32
43	MT470102.1	France	45.37	0.26	49.32
44	MT709104.1	France	46.26	0.27	50.43
45	MT709105.1	France	45.38	0.26	49.32
46	MT470106.1	France	45.36	0.26	49.32
47	MT066156.1	Italy	50.98	0.36	56.31
48	MT525950.1	Italy	45.38	0.26	49.32
49	MT622321.1	Italy	45.37	0.26	49.32
50	MT682732.1	Italy	45.39	0.26	49.32
51	MT748758.1	Italy	45.39	0.26	49.32
52	MT890669.1	Italy	45.38	0.26	49.32
53	MT635445.1	Russia	45.38	0.26	49.32
54	MT637143.1	Russia	45.37	0.26	49.32
55	MT890462.1	Russia	45.38	0.26	49.32
56	MT198652.2	Spain	46.43	0.28	50.51
57	MT233519.1	Spain	46.1	0.27	49.92
58	MT233522.1	Spain	52.06	0.36	56.09
59	MT233523.1	Spain	45.36	0.26	49.25
60	MT292569.1	Spain	45.38	0.26	49.32
Mean			45.80	0.27	49.81
Std			1.27	0.02	1.46

Supplementary Table S3: *Neutrality plot (GC3 and GC12) value comparison of all the sixty strains of SARS-CoV-2*

**S. No**	**Accession Number**	**Country**	**GC3s**	**GC12**	**S. No**	**Accession Number**	**Country**	**GC3s**	**GC12**
1	MT500122.1	Pakistan	0.26	42.73	31	MT890210.1	Ghana	0.27	42.33
2	MT879619.1	Pakistan	0.26	42.76	32	MT890211.1	Ghana	0.29	40.82
3	MT262993.1	Pakistan	0.26	42.79	33	MT890230.1	Ghana	0.30	40.89

4	MT240479.1	Pakistan	0.26	42.76	34	MT890235.1	Ghana	0.27	42.41
5	MT135042.1	China	0.26	42.86	35	MT890236.1	Ghana	0.32	39.61
6	MT135043.1	China	0.26	42.84	36	MT499215.1	Tunisia	0.26	42.77
7	MT121215.1	China	0.26	43.14	37	MT499217.1	Tunisia	0.26	42.76
8	MN996529.1	China	0.26	42.82	38	MT499218.1	Tunisia	0.26	42.73
9	NC_045512.2	China	0.26	62.11	39	MT499219.1	Tunisia	0.26	42.76
10	MT799974.1	India	0.26	42.73	40	MT499220.1	Tunisia	0.26	42.76
11	MT607244.1	India	0.26	42.75	41	MT470100.1	France	0.26	42.77
12	MT635856.1	India	0.26	42.78	42	MT470101.1	France	0.26	42.75
13	MT457402.1	India	0.26	38.88	43	MT470102.1	France	0.26	42.76
14	MT012098.1	India	0.26	42.85	44	MT709104.1	France	0.27	41.90
15	MT509506.1	India	0.26	35.58	45	MT709105.1	France	0.26	42.76
16	MT007544.1	Australia	0.26	42.73	46	MT470106.1	France	0.26	42.76
17	MT745608.1	Australia	0.26	42.74	47	MT066156.1	Italy	0.36	37.28
18	MT745611.1	Australia	0.26	66.19	48	MT525950.1	Italy	0.26	42.75
19	MT745629.1	Australia	0.26	42.74	49	MT622321.1	Italy	0.26	42.75
20	MT745630.1	Australia	0.30	39.95	50	MT682732.1	Italy	0.26	42.77
21	MT126808.1	Brazil	0.26	42.81	51	MT748758.1	Italy	0.26	42.79
22	MT835026.1	Brazil	0.26	42.78	52	MT890669.1	Italy	0.26	42.75
23	MT835027.1	Brazil	0.26	42.77	53	MT635445.1	Russia	0.26	42.78
24	MT835383.1	Brazil	0.26	42.78	54	MT637143.1	Russia	0.26	42.74
25	MT844030.1	Brazil	0.26	42.76	55	MT890462.1	Russia	0.26	42.75
26	MT163719.1	USA	0.26	42.79	56	MT198652.2	Spain	0.28	41.87
27	MT106054.1	USA	0.26	42.82	57	MT233519.1	Spain	0.27	42.42
28	MT325622.1	USA	0.26	42.73	58	MT233522.1	Spain	0.36	37.30
29	MT325627.1	USA	0.26	42.78	59	MT233523.1	Spain	0.26	42.84
30	MT325598.1	USA	0.26	42.72	60	MT292569.1	Spain	0.26	42.77
Mean								0.27	42.91
Std								0.02	4.27

Supplementary Table S4: *CAI values of sixty strains of SARS-CoV-2 with host humans.*

**S. No**	**Accession Number**	**Country**	**CAI Homo-sapiens**	**S. No**	**Accession Number**	**Country**	**CAI Homo-sapiens**
1	MT500122.1	Pakistan	0.69	32	MT890211.1	Ghana	0.71
2	MT879619.1	Pakistan	0.69	33	MT890230.1	Ghana	0.70
3	MT262993.1	Pakistan	0.69	34	MT890235.1	Ghana	0.69
4	MT240479.1	Pakistan	0.69	35	MT890236.1	Ghana	0.71
5	MT135042.1	China	0.69	36	MT499215.1	Tunisia	0.69
6	MT135043.1	China	0.69	37	MT499217.1	Tunisia	0.69

7	MT121215.1	China	0.69	38	MT499218.1	Tunisia	0.69
8	MN996529.1	China	0.69	39	MT499219.1	Tunisia	0.69
9	NC_045512.2	China	0.69	40	MT499220.1	Tunisia	0.69
10	MT799974.1	India	0.69	41	MT470100.1	France	0.70
11	MT607244.1	India	0.69	42	MT470101.1	France	0.70
12	MT635856.1	India	0.69	43	MT470102.1	France	0.70
13	MT457402.1	India	0.69	44	MT709104.1	France	0.70
14	MT012098.1	India	0.69	45	MT709105.1	France	0.70
15	MT509506.1	India	0.69	46	MT470106.1	France	0.70
16	MT007544.1	Australia	0.69	47	MT066156.1	Italy	0.69
17	MT745608.1	Australia	0.69	48	MT525950.1	Italy	0.69
18	MT745611.1	Australia	0.69	49	MT622321.1	Italy	0.69
19	MT745629.1	Australia	0.69	50	MT682732.1	Italy	0.69
20	MT745630.1	Australia	0.70	51	MT748758.1	Italy	0.69
21	MT126808.1	Brazil	0.69	52	MT890669.1	Italy	0.69
22	MT835026.1	Brazil	0.69	53	MT635445.1	Russia	0.69
23	MT835027.1	Brazil	0.69	54	MT637143.1	Russia	0.69
24	MT835383.1	Brazil	0.69	55	MT890462.1	Russia	0.69
25	MT844030.1	Brazil	0.69	56	MT198652.2	Spain	0.73
26	MT163719.1	USA	0.69	57	MT233519.1	Spain	0.73
27	MT106054.1	USA	0.69	58	MT233522.1	Spain	0.73
28	MT325622.1	USA	0.69	59	MT233523.1	Spain	0.73
29	MT325627.1	USA	0.69	60	MT292569.1	Spain	0.73
Standard deviation				0.01055549			
Mean				0.70			

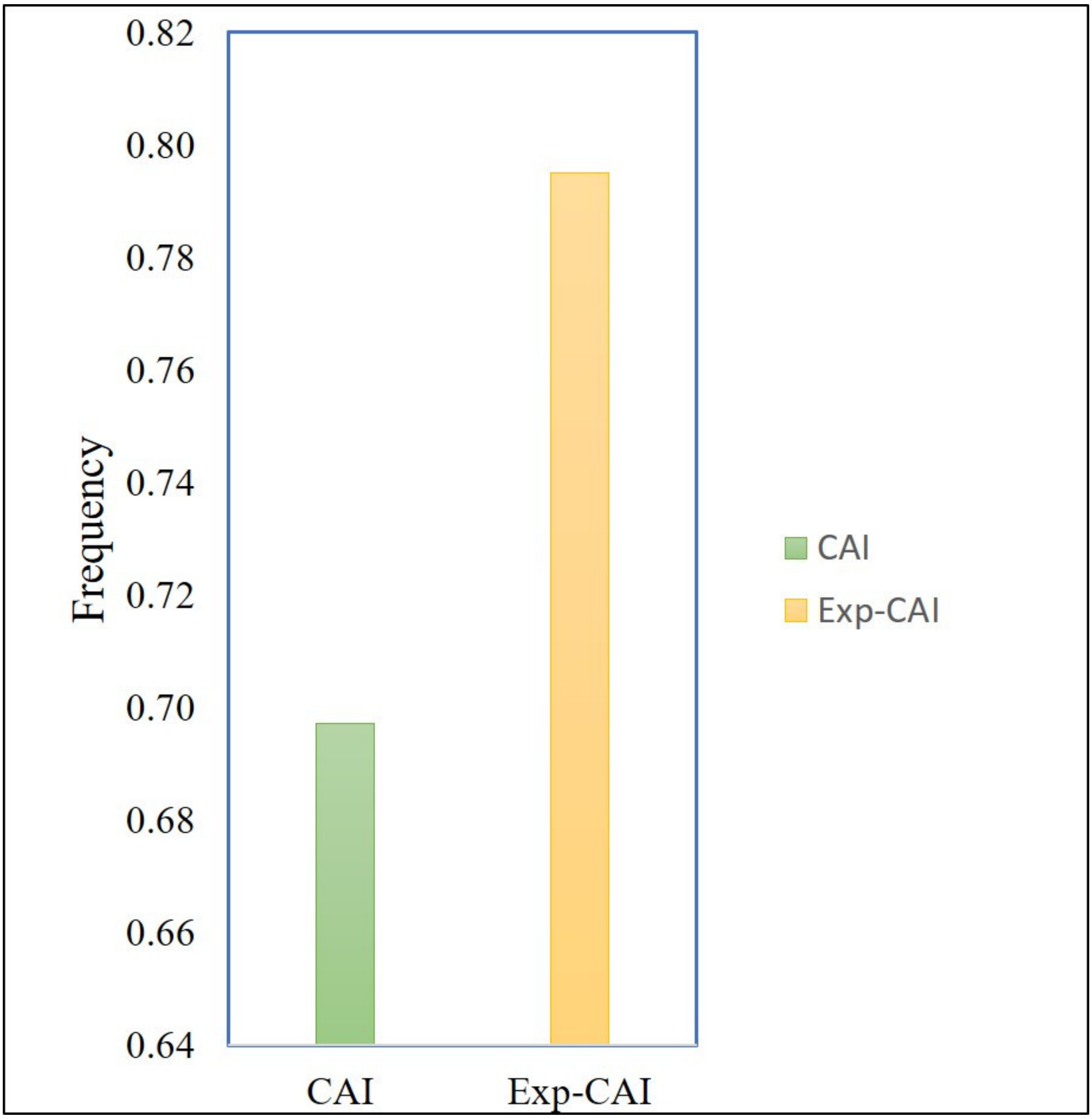

Figure S1: *Mean CAI value and Expected CAI value of SARS-CoV-2 strains against its host Humo sapiens.*
